# Hepatic arterial infusion chemotherapy combined with apatinib plus camrelizumab for advanced hepatocellular carcinoma with type Vp4 portal vein tumor thrombosis: a multicenter propensity score-matching analysis

**DOI:** 10.3389/fimmu.2026.1742116

**Published:** 2026-02-17

**Authors:** Shaoteng Wu, Feifeng Qiu, Fengtao Zhang, Pingkang Chen, Xiang Zheng, Qiming Wei, Haiming Zhang, Cheng Qian, Ankang Shu, Ming Li, Dejun Xiong, Sheng Zhong

**Affiliations:** 1 Department of Tumor and Vascellum Intervention, DongGuan Tungwah Hospital, DongGuan, Guangdong, China; 2 Department of Interventional Surgery Room, The First Affiliated Hospital of Jinan University, Guangzhou, China; 3 Vascular Interventional Surgery, Shenzhen Nanshan People’s Hospital (Affiliated Nanshan Hospital of Shenzhen University), Shenzhen, Guangdong, China; 4 Department of Interventional Therapy, Zhuhai People’s Hospital (Zhuhai Clinical Medical College of Jinan University), Zhuhai, Guangdong, China; 5 Department of Interventional, The Second Affiliated Hospital of Guangzhou University of Chinese Medicine, Guangzhou, Guangdong, China; 6 Department of Radiology, the First Affiliated Hospital of Guangdong Pharmaceutical University, Guangzhou, Guangdong, China; 7 School of Science and Engineering, The Chinese University of Hong Kong Shenzhen, Shenzhen, Guangdong, China

**Keywords:** apatinib, camrelizumab, hepatic arterial infusion chemotherapy, hepatocellular carcinoma, portal vein main trunk invasion

## Abstract

**Background:**

Portal vein main trunk invasion is a serious and difficult complication of hepatocellular carcinoma (HCC), with extremely poor prognosis and limited treatment options. The traditional standard sorafenib has a limited efficacy. The combination of hepatic arterial infusion chemotherapy (HAIC) with camrelizumab and apatinib has shown satisfactory efficacy in previously advanced HCC. Therefore, this approach has potential advantageous survival benefits for HCC with invasion of the portal vein main trunk.

**Methods:**

A retrospective review was conducted on the clinical data of advanced HCC patients with type Vp4 portal vein invasion who received HAIC combined with apatinib and camrelizumab (HAICAC group) or HAIC alone (HAIC group) treatment in four medical centers from June 2016 to December 2023. Propensity score matching was employed to balance the baseline differences between the groups. The overall survival, progression-free survival, objective response rate and disease control rate were compared between the groups.

**Results:**

Following PSM, the HAICAC regimen demonstrated significantly superior clinical outcomes, with median OS (24.1 versus 7.2 months) and PFS (7.0 versus 4.3 months) significantly exceeding those of HAIC monotherapy (all P<0.001). The combination therapy also exhibited markedly improved tumor response rates, achieving superior objective response rates for both intrahepatic lesions (75.6% versus 31.4%, P<0.001) and PVTT (60.5% versus 17.4%, P<0.001). While the HAICAC group showed a higher incidence of immune-related adverse events compared to the HAIC group, all events were manageable and no grade 5 toxicities occurred.

**Conclusion:**

For HCC with Vp4 type PVTT, the combination regimen of HAIC plus apatinib and camrelizumab demonstrates promising efficacy in reducing both intrahepatic tumor burden and thrombus progression, representing a potentially viable treatment approach with an acceptable safety profile.

## 1 Introduction

Hepatocellular carcinoma (HCC) represents a highly heterogeneous primary liver malignancy, ranking as the fourth most prevalent cancer and the third leading cause of cancer-related mortality worldwide (1). Portal vein tumor thrombosis (PVTT) is a particularly formidable complication of HCC, manifests in approximately 10%-60% of patients at initial diagnosis (2). Regrettably, the conventionally recommended sorafenib demonstrates limited efficacy in managing HCC with PVTT, particularly for portal vein main trunk invasion (Vp4 type). This advanced disease state portends an exceptionally dismal prognosis, with a median overall survival (OS) of merely 6 months (3).

Transarterial chemoembolization (TACE) has been demonstrated to exhibit limited efficacy in managing PVTT and its application in Vp4 PVTT remains highly controversial (4). In contrast, hepatic arterial infusion chemotherapy (HAIC) represents a locoregional therapeutic approach that delivers chemotherapeutic agents directly into tumor sites via an indwelling microcatheter, thereby enhancing local drug concentration while significantly mitigating systemic toxicity. The pivotal FOHAIC-1 trial established the superiority of HAIC over sorafenib in managing advanced HCC, demonstrating a median OS of 13.4 months with HAIC monotherapy which statistically significant improvement compared to the 8.4 months of sorafenib, even in a cohort with high prevalence of macrovascular invasion (5). Consequently, HAIC has emerged as a promising primary locoregional treatment option for PVTT (6). Notably, the Japanese Society of Hepatology (JSH) guidelines have endorsed HAIC as one of the preferred therapeutic strategies for HCC with portal vein involvement (7).

The past decade has witnessed remarkable advancements in systemic therapy for HCC. The groundbreaking IMbrave150 trial first demonstrated the compelling survival benefits of combining atezolizumab with bevacizumab in unresectable HCC, establishing this dual immunotherapy-antiangiogenesis regimen as a landmark therapeutic strategy (8). According to the Barcelona Clinic Liver Cancer (BCLC) staging system, the presence of PVTT, irrespective of subtype or other tumor characteristics and automatically classifies HCC as advanced-stage disease, for which the atezolizumab-bevacizumab combination is recommended as the first-line standard treatment. However, updated data from the IMbrave150 trial showed that HCC patients with Vp4 PVTT treated with this combination achieved a median OS of only 7.6 months (9). Subsequently, the CARES-310 trial reported even more impressive outcomes with apatinib plus camrelizumab, achieving a median OS of 22.1 months, surpassing the results of the IMbrave150 regimen and suggesting a potential survival advantage for this novel combination in advanced HCC (10). In advanced HCC with PVTT, a particularly aggressive and treatment-resistant subtype, HAIC based combination therapies have emerged as a highly promising strategy. Recent TRIPLET trial evaluating HAIC combined with apatinib and camrelizumab in BCLC stage C HCC (with 71.8% of PVTT patients) demonstrated exceptional efficacy, including a median progression-free survival (PFS) of 10.38 months and an objective response rate (ORR) of 77.1%, indicating that this regimen can achieve meaningful tumor regression and disease control even in this rapidly progressing subset (11). Thus, for HCC with Vp4 PVTT, a condition historically associated with an exceptionally dismal prognosis and this innovative multimodal approach may represent a paradigm shift, offering unprecedented survival benefits for these patients with exceedingly poor prognosis.

Currently, Vp4 PVTT remains a thorny problem and faces severe challenges. As of now, the efficacy of HAIC combined with apatinib and camrelizumab in the treatment of HCC with Vp4 PVTT has not been clearly determined. Therefore, this multicenter retrospective study aims to investigate the safety and efficacy of HAIC combined with apatinib and camrelizumab in HCC patients with Vp4 PVTT.

## 2 Methods

### 2.1 Study population

This multicenter, retrospective study complied with the ethical principles of the *Declaration of Helsinki (1975)* and was approved by the Institutional Review Board of Shenzhen Nanshan People’s Hospital (Approval number: Ky-2024-042604). HAIC treatment related written informed consent was obtained from all patients. The clinical data of HCC patients with Vp4 PVTT received HAIC combined with apatinib and camrelizumab or HAIC alone treatment at four Chinese medical centers from June 2016 to December 2023 were retrospectively collected and analyzed. The inclusion criteria were as follows: (1) HCC diagnosis confirmed according to the *European Association for the Study of the Liver (EASL) guidelines* or via liver histopathological biopsy; (2) Confirm the diagnosis of Vp4 PVTT through CT or MRI; (3) Age>18 years; (4) Liver function (Child-Pugh class A–B or ALBI grade 1–2); (5) Eastern Cooperative Oncology Group (ECOG) performance status<2. Exclusion criteria comprised: (1) Prior antitumor therapy before HAIC initiation; (2) Incomplete clinical records; (3) Loss to follow-up for >6 months; (4) Concurrent other malignancies.

### 2.2 Treatment procedure and follow-up

The HAIC procedure is detailed in **Supplementary Material 1**. The systemic therapy was initiated within 3 days from the start of the first HAIC procedure, consisting of apatinib administered orally at 250mg daily and camrelizumab given at 200mg (for patients >50kg) or 3mg/kg (for patients <50kg) every 14 days. Treatment discontinuation occurred upon withdrawal of informed consent or development of intolerable adverse reactions. Dose reduction or temporary interruption of apatinib, as well as temporary withholding of camrelizumab were permitted for grade 3–4 treatment-related adverse events, with resumption upon symptom resolution. Second-line therapy was initiated and follow-up continued in cases of disease progression or intolerable treatment-related toxicity.

Follow-up scheme: Patients were followed up monthly during the first treatment cycle and subsequently at three-month intervals. Follow-up evaluations included comprehensive assessments of liver function, serum AFP levels, complete blood counts, and contrast-enhanced abdominal CT or MRI scans. PET-CT examinations were performed when clinically indicated for systemic evaluation.

### 2.3 Survival outcomes and tumor response

This study established OS as the primary endpoint, measured from the date of histologically confirmed HCC diagnosis to either the last follow-up or death from any cause. The secondary endpoint was PFS which calculated from initial diagnosis to first radiological confirmation of disease progression (PD) based on modified Response Evaluation Criteria in Solid Tumors version 1.1 (mRECIST 1.1). Two board-certified radiologists independently assessed tumor response and PVTT regression according to mRECIST 1.1 criteria, categorizing outcomes as complete response (CR), partial response (PR), stable disease (SD), or progressive disease (PD). Additional efficacy measures included the objective response rate (ORR; CR+PR proportion) for both intrahepatic lesions and PVTT, along with the disease control rate (DCR; CR+PR+SD cases). All treatment-related adverse events were evaluated following Common Terminology Criteria for Adverse Events version 5.0 (CTCAE v5.0) standards.

### 2.4 Propensity score matching

Propensity scores were estimated using a multivariable logistic regression model that included the following covariates: age, gender, ECOG performance status, HBV infection status, liver cirrhosis background, Child-Pugh classification, ALBI grade, AFP level, maximal tumor diameter, tumor multiplicity, and extrahepatic metastasis. A 1:1 nearest matching algorithm without replacement was then applied. Caliper width was set at 0.02. To ensure the robustness of matching, the order of cases was randomized prior to matching. Matching was performed using the MatchIt package in R software (version 4.3.1). The balance plot after propensity score matching is shown in **Supplementary Figure 1**.

### 2.5 Statistical analysis

Statistical analyses were performed using R software (RStudio version 4.3.1) and SPSS 26.0 (IBM Corp., NY, USA). Continuous variables were expressed as mean ± standard deviation or median (interquartile range) based on normality testing (Shapiro-Wilk test) and compared using Student’s t-test or Mann-Whitney U test, as appropriate. Categorical variables were analyzed by χ² test or Fisher’s exact test. Survival outcomes were evaluated via Kaplan-Meier methodology with log-rank testing and survival curves were generated. Univariate and multivariate analyses were conducted using Cox proportional hazards regression models to identify prognostic factors and perform subgroup analyses. Covariates with P<0.1 demonstrating potential significance in univariate analysis were incorporated into the multivariate model.

A two-tailed P value < 0.05 was considered statistically significant.

## 3 Results

### 3.1 Patient characteristics

**Figure 1:**
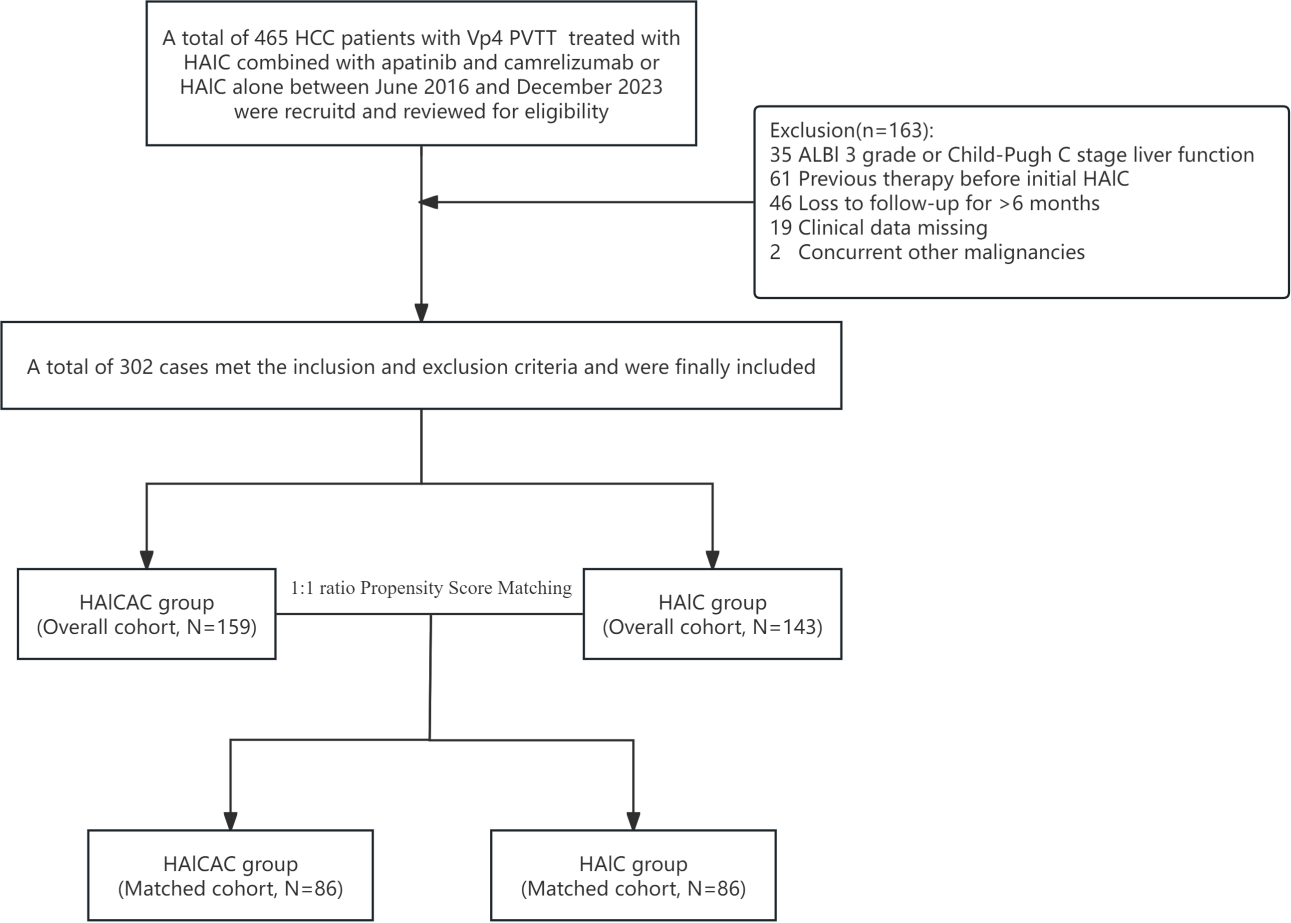
Flowchart of the patients selection process for this study.

**Figure 2:**
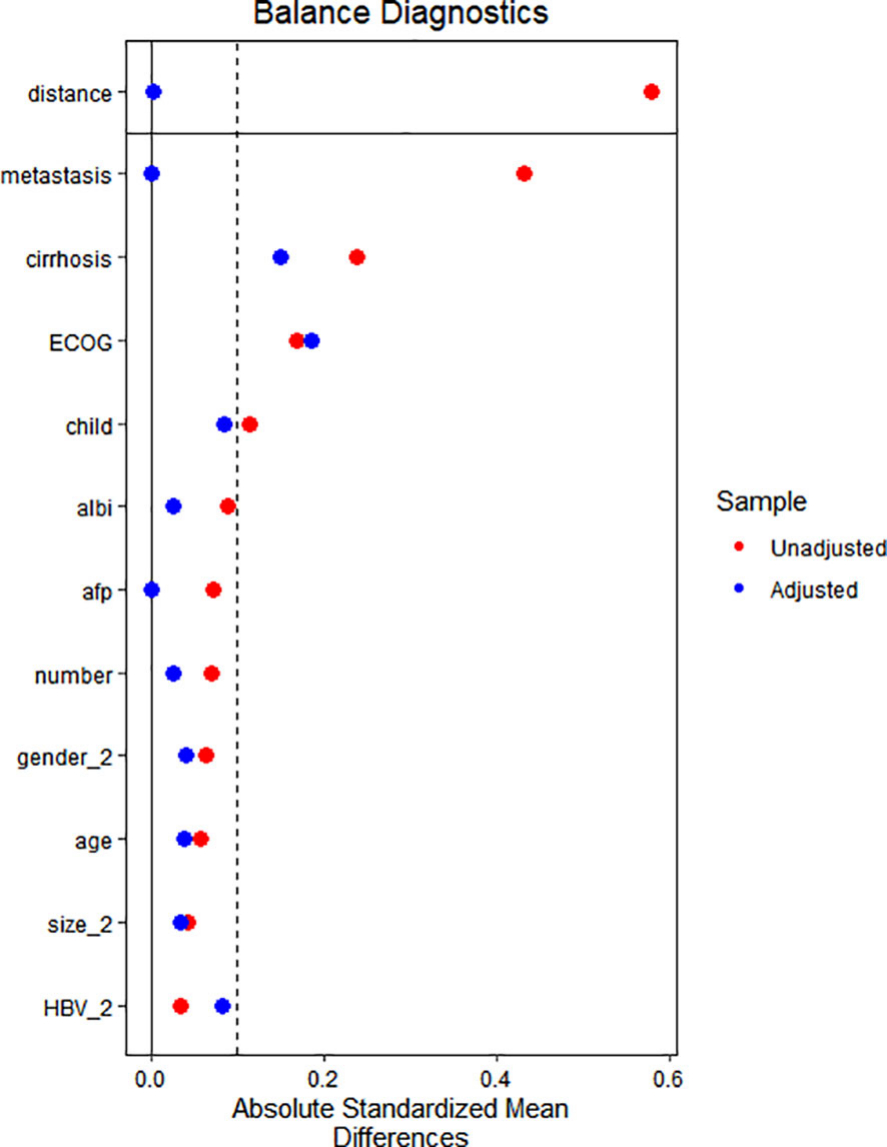
Propensity score matching(PSM) balance plot.

Following a comprehensive eligibility assessment, a total of 159 HCC patients with portal vein main trunk invasion (Vp4 type) who underwent HAIC combined with apatinib plus camrelizumab therapy and 143 patients receiving HAIC monotherapy were ultimately enrolled in the study. The patient enrollment flowchart is presented in **Figure 1**. Baseline characteristic comparisons revealed that the HAIC monotherapy cohort exhibited significantly higher proportions of concomitant liver cirrhosis and extrahepatic metastases. 1:1 ratio PSM was employed to mitigate intergroup baseline disparities, resulting in optimal matching of all demographic parameters and yielding a final PSM cohort comprising 172 cases. The balance plot after propensity score matching is shown in **Figure 2**. The enrolled patients predominantly presented with HBsAg positivity, liver cirrhosis and high tumor burden (characterized by large tumor diameter and multinodular involvement). The mean tumor diameters were 12.1cm in the combination therapy group and 11.3cm in the monotherapy group, respectively. A comprehensive summary of all baseline characteristics is provided in **Table 1**.

**Table 1:** Baseline characteristics of the study patients before and after PSM.

	Overall cohort				PSM cohort			
	HAICAC group (n=159)	HAIC group (n=143)	P value	SMD	HAICAC group (n=86)	HAIC group (n=86)	P value	SMD
Age^a^	46.7 ± 11.8y	47.3 ± 10.9	0.478	0.059	47.0 ± 11.6	47.6 ± 9.9	0.787	0.041
≤65y	150(94.3%)	132 (92.3%)			79(91.9%)	78(90.7%)		
>65y	9(5.7%)	11(7.7%)			7(8.1%)	8(9.3%)		
Gender			0.873	0.062			0.787	0.043
Male	138(86.8%)	125(88.7%)			78(90.7%)	79(91.9%)		
Female	21(13.2%)	18(11.3%)			8(9.3%)	7(8.1%)		
ECOG PS			0.201	0.189			0.418	0.251
0	147(92.5%)	126(88.1%)			77(89.5%)	80(93.0%)		
1	12(7.5%)	17(11.9%)			9(10.5%)	6(7.0%)		
HBsAg			0.998	0.035			0.846	0.084
Presence	139(87.4%)	125(87.4%)			69 (80.2%)	70(81.4%)		
Absence	20(12.6%)	18(12.6%)			17 (19.8%)	16(18.6%)		
Cirrhosis			0.008	0.215			0.816	0.147
Presence	126(79.2%)	130(90.9%)			75(87.2%)	76(88.4%)		
Absence	33(20.8%)	13(9.1%)			11(12.8%)	10(11.6%)		
Child-Pugh grade			0.354	0.107			0.798	0.104
A	138(86.8%)	129(90.2%)			77(89.5%)	78(90.7%)		
B	21(13.2%)	14(9.8%)			9(10.5%)	8(9.3%)		
ALBI grade			0.447	0.088			0.535	0.026
1	68(42.8%)	55(38.5%)			37(43.0%)	33(38.4%)		
2	91(57.2%)	88(61.5%)			49(57.0%)	53(61.6%)		
AFP			0.923	0.070			1.000	<0.001
≤400ng/L	47(29.6%)	43(30.1%)			26(30.2%)	26(30.2%)		
>400ng/L	112(70.4%)	100(69.9%)			60(69.8%)	60(69.8%)		
ALB^b^(g/L)	37.9(35.0-45.8)	39.3(36.2-43.7)	0.267	–	38.6(35.5-44.6)	39.2(36.0-45.1)	0.537	–
ALT^b^(U/L)	44.2(32.5-64.9)	45.3(31.2-69.0)	0.534	–	43.7(31.0-66.9)	44.2(31.6-68.7)	0.438	–
AST^b^(U/L)	76.8(49.5-127.1)	77.2(50.2-126.3)	0.734	–	77.9(49.5-130.2)	77.1(51.2 -127.6)	0.537	–
TBIL^b^(umol/l)	17.6(12.7-25.1)	17.3(12.2-25.3)	0.751	–	18.1(11.9-23.7)	17.8(11.8-24.3)	0.934	–
Tumor diameter	12.1 ± 3.5cm	11.3 ± 3.8cm	0.785	0.041	12.0 ± 3.1cm	11.5 ± 3.2cm	0.149	0.037
≤7cm	24(15.1%)	20(14.0%)			4(4.7%)	9(10.5%)		
>7cm	135(84.9%)	123(86.0%)			82(95.3%)	77(89.5%)		
Tumor number			0.272	0.069			0.624	0.025
1-3	58(36.5%)	43(30.1%)			29(33.7%)	26(30.2%)		
>3	101(63.5%)	100(69.9%)			57(66.3%)	60(69.8%)		
Extrahepatic metastasis			0.004	0.426			0.878	<0.001
Presence	65(40.9%)	83(58.0%)			39(45.3%)	40(46.5%)		
Absence	94(59.1%)	60(42.0%)			47(54.7%)	46(53.5%)		

P-value < 0.05 indicated a significant difference.

a. Data are means ± standard deviations.

b. Data are medians, with interquartile ranges in parentheses.

PSM, Propensity Score Matching; HAICAC, Hepatic arterial infusion chemotherapy combined with apatinib plus camrelizumab therapy; HAIC, Hepatic arterial infusion chemotherap; SMD, standardized mean differences. ECOG PS, Eastern Cooperative Oncology Group Performance Status; HBsAg, Hepatitis B surface antigen; ALBI, Albumin-bilirubin; AFP, α-fetoprotein; ALB,: Albumin; ALT, alanine aminotransferase; AST, aspartate aminotransferase; TBIL, total bilirubin; PVTT: Portal vein tumor thrombosis.

### 3.2 Survival outcomes

**Figure 3:**
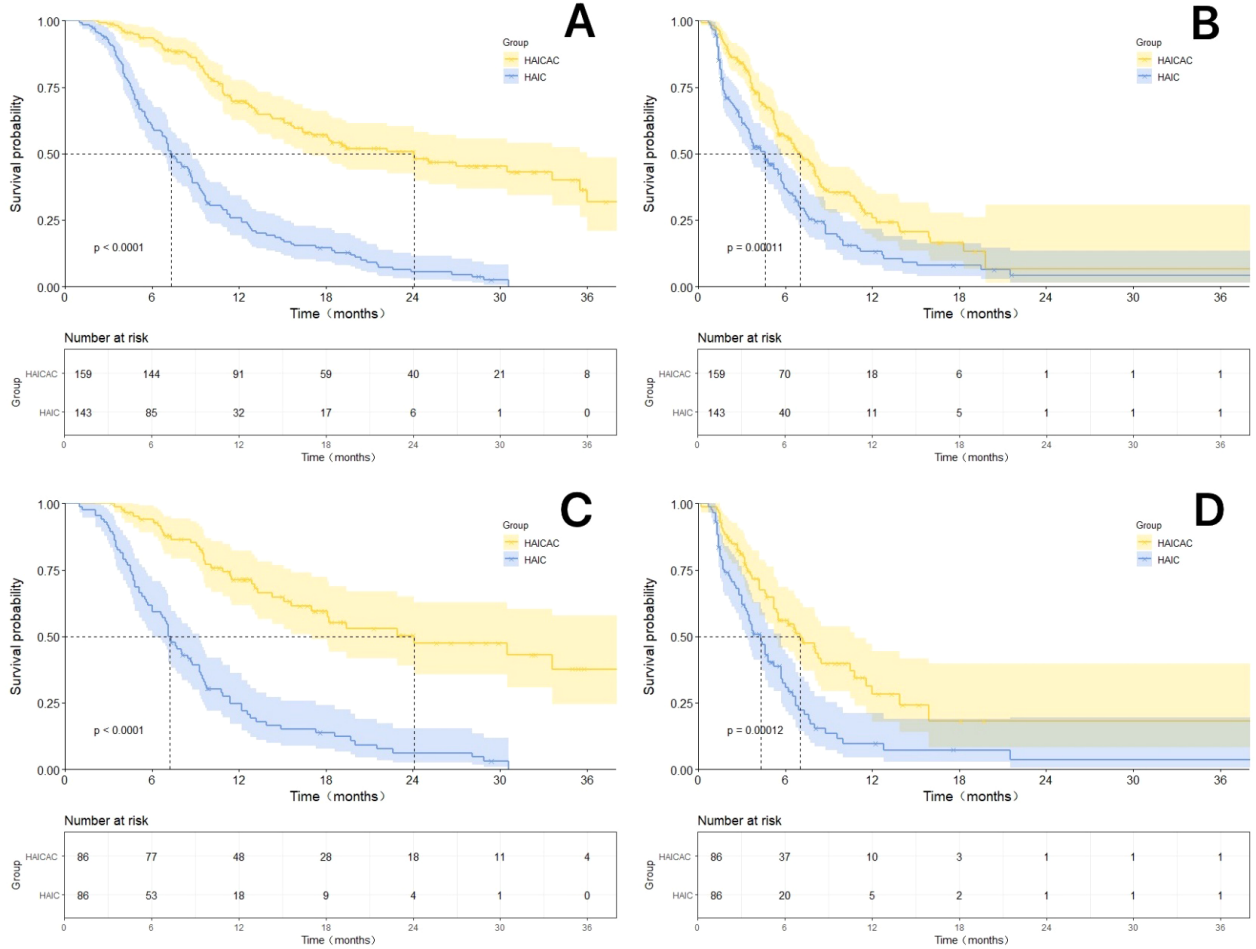
The Kaplan-Meier survival curves for the HAICAC group and the HAIC group with or without propensity score matching (PSM) adjustment. **(A)** The Kaplan-Meier curves comparing the overall survival between the HAICAC group and the HAIC group without PSM-adjusted; **(B)** The Kaplan-Meier curves comparing the overall survival between the HAICAC group and the HAIC group without PSM-adjusted; **(C)**. Comparison of PSM-adjusted overall survival between the HAICAC group and HAIC groups; **(D)** Comparison of PSM-adjusted progression-free survival between the HAICAC group and HAIC groups.

Prior to propensity matching, with a mean follow-up of 21.3 months, the HAICAC group demonstrated an encouraging median OS of 24.1 months, which was significantly superior to the 7.3 months observed in the HAIC monotherapy group (HR: 0.30; 95%CI: 0.23-0.40; P<0.001). Regarding PFS, the HAICAC regimen showed a superior median PFS of 7.0 months compared to 4.6 months in the HAIC group (HR: 0.66; 95%CI: 0.50-0.86; P<0.001). After PSM, the HAICAC group maintained similarly significant advantages in both OS (24.1 months vs 7.2 months; HR: 0.30; 95%CI: 0.20-0.44; P<0.001) and PFS (7.0 months vs 4.3 months; HR: 0.61; 95%CI: 0.43-0.89; P<0.001). The Kaplan-Meier curves before and after PSM are presented in **Figure 3**.

### 3.3 Tumor response

The best tumor responses for both intrahepatic lesions and PVTT before and after matching are presented in **Table 2**. In the matched cohorts, the HAICAC group achieved an encouraging 75.6% ORR and 94.2% DCR for intrahepatic tumors, compared to merely 31.4% ORR and 59.3% DCR in the HAIC group (P<0.05 for overall response, ORR and DCR). Regarding PVTT regression, the combination therapy yielded substantially higher response rates (60.5% of ORR and 90.7% of DCR) than monotherapy (17.4% ORR and 59.3% DCR, P<0.001).

**Table 2:** The best tumor and PVTT response before and after propensity score matching.

Best Response	Overall cohort			PSM cohort		
	HAICAC group(n=159)	HAIC group (n=143)	P value	HAICAC group(n=86)	HAIC group (n=86)	P value
Intrahepatic tumor			< 0.001			0.003
CR	12(7.5%)	1(0.7%)		7(8.1%)	0(0%)	
PR	108(67.9%)	44(30.8%)		58(67.4%)	27(31.4%)	
SD	31(19.5%)	36(25.2%)		16(18.6%)	24(27.9%)	
PD	8(5.0%)	62(43.4%)		5(5.8%)	35(40.7%)	
ORR	75.5%(120/159)	31.5%(45/143)	< 0.001	75.6%(65/86)	31.4%(27/86)	< 0.001
DCR	95.0%(151/159)	56.6%(81/143)	< 0.001	94.2%(81/86)	59.3%(51/86)	< 0.001
PVTT						
CR	8(5.8%)	0(0%)	< 0.001	6(7.0%)	0(0%)	< 0.001
PR	85(53.4%)	26(18.2%)		46(53.5%)	15(17.4%)	
SD	53(33.3%)	53(37.1%)		26(30.2%)	36 (41.9%)	
PD	13(8.2%)	64(44.8%)		8(9.3%)	35(40.7%)	
ORR	58.4%(93/159)	18.2%(26/143)	< 0.001	60.5%(52/86)	17.4%(15/86)	< 0.001
DCR	91.8%(146/159)	55.2%(79/143)	< 0.001	90.7%(78/86)	59.3%(51/86)	< 0.001

RECIST, Response evaluation criteria in solid tumors; mRECIST, Modified response evaluation criteria in solid tumors; HAICLT, Hepatic arterial infusion chemotherapy combined with apati**n**ib plus camrelizumab therapy; HAIC, Hepatic arterial infusion chemotherap; CR, Complete response; PR, partial response; SD, Stable disease; PD, Progressive disease; ORR, Objective response rate; DCR, Disease control rate; PVTT, protal vein tumor thombsis.

### 3.4 Univariate and multivariate analysis

The univariate and multivariate analyses for OS and PFS are detailed in **Table 3**. Univariate and multivariate analyses identified ECOG PS 0, ALBI grade 1 liver function and treatment regimen as significant prognostic factors for superior OS. Similarly, ECOG PS 0, low AFP levels, absence of extrahepatic metastasis, and treatment regimen emerged as independent risk factors for prolonged PFS.

**Table 3:** Risk factors for overall survival and progression-free survival based on uni- and multivariate analysis.

Factors	Overall survival				Progression-free survival			
	Univariate analysis P value	Multivariate analysis			Univariate analysis P value	Multivariate analysis		
		HR	95%CI	P value		HR	95%CI	P value
Gender	**0.070**	1.30	0.75-2.21	0.355	0.348	–	–	–
Male								
Female								
Age	0.153	–	–	0.	0.967	–	–	–
≤65y								
>65y								
ECOG PS	**0.002**	0.76	0.13-1.68	**<0.001**	**0.035**	0.51	0.24-0.87	**0.004**
0								
1								
HBsAg	**0.001**	1.10	0.76-1.60	0.598	**0.059**	0.96	0.69-1.34	0.816
Presence								
Absence								
Crrihosis	0.545	–	–	–	0.139	–	–	–
Presence								
Absence								
Child-Pugh grade	0.360	–	–	–	0.183	–	–	–
A								
B								
ALBI grade	**0.031**	1.41	1.06-1.88	**0.020**	0.609	–	–	–
1								
2								
AFP	0.831	–	–	–	**0.025**	0.37	0.14-0.69	**0.005**
≤400ng/mL								
>400ng/mL								
Large tumor diameter	**0.016**	0.74	0.50-1.11	0.147	0.752			–
≤7cm								
>7cm								
Tumor number	**0.099**	1.30	0.96-1.77	0.093	0.437	–	–	–
1-3								
>3								
Extrahepatic metastasis	0.736	–	–	–	**0.024**	0.32	0.21-0.56	**< 0.001**
Presence								
Absence								
Treatment regimen	**<0.001**	0.22	0.15-0.31	**< 0.001**	**< 0.001**	0.48	0.35-0.66	**< 0.001**
HAICAC								
HAIC								

HR, Hazard ratios; CI, Confidence interval; ECOG PS, Eastern cooperative oncology group performance status; HBsAg, Hepatitis B surface antigen; ALBI, Albumin-bilirubin ratio; AFP,α-fetoprotein; PVTT, Portal vein tumor thrombosis; HAICLT, Hepatic arterial infusion chemotherapy combined with apatinib plus camrelizumab therapy; HAIC, Hepatic arterial infusion chemotherapy. Bold indicates statistical significance level at p-value < 0.1.

### 3.5 Subgroup analysis

**Figure 4:**
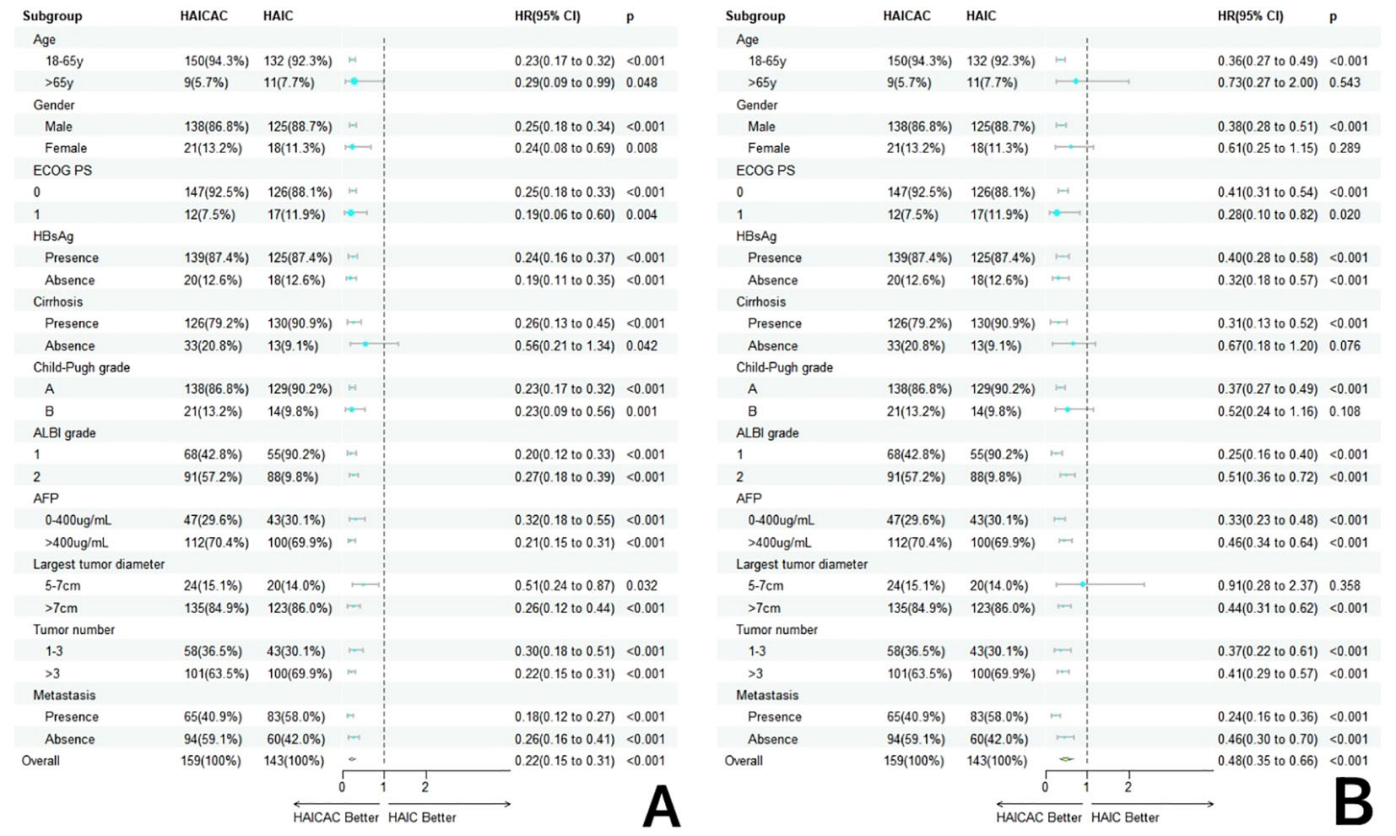
Forestplot based on overall survival **(A)** and progression-free survival **(B)** of each subgroup.

**Figure 5:**
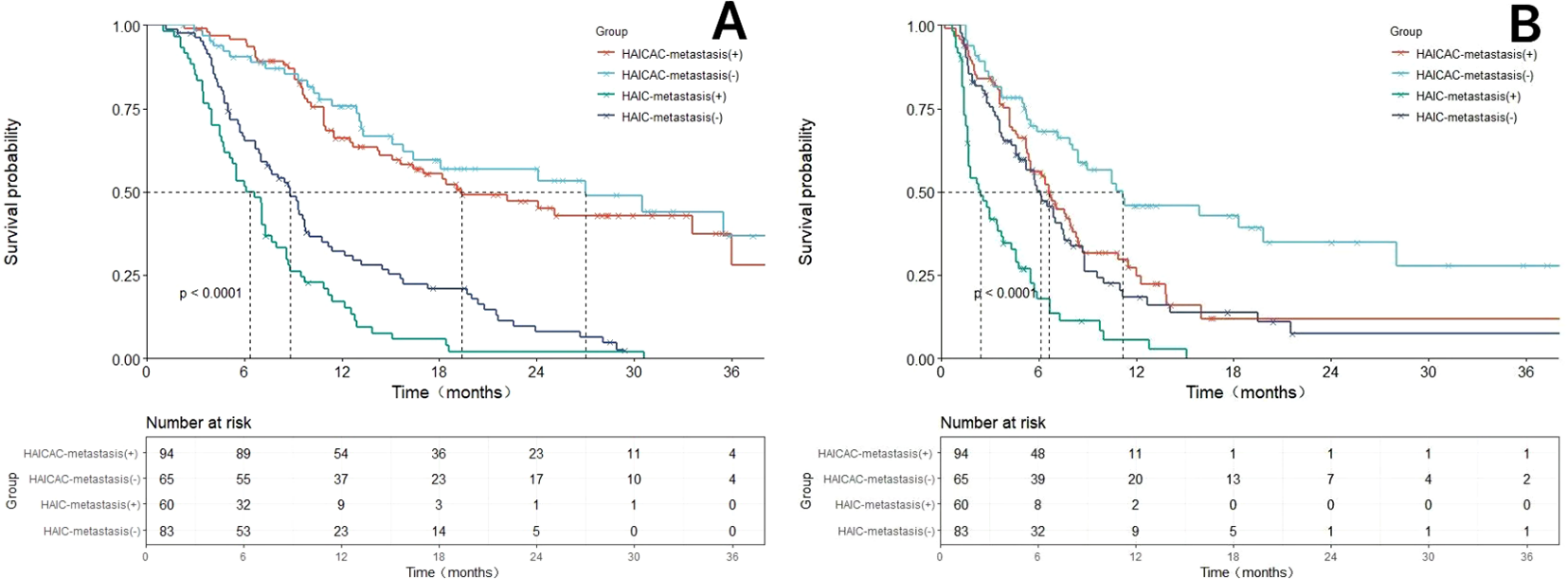
Comparison of overall survival (OS) and progression-free survival (PFS) before and after propensity score matching (PSM) adjustment based on the HAICAC group and HAIC group with or without merging hepatic extrahepatic spread subgroups. **(A)** Comparison of OS between the HAICAC group and HAIC group in the metastatic and non-metastatic subgroups. **(B)** Comparison of PFS between the HAICAC group and HAIC group in the metastatic and non-metastatic subgroups.

Forest plots (**Figure 4**) were generated to evaluate treatment outcomes across different subgroups. The triple-therapy regimen demonstrated superior OS benefits in most analyzed subgroups. However, certain subgroups, including elderly patients, females, non-cirrhotic individuals, Child-Pugh B liver function and small nodular tumors, did not achieve statistically significant improvements, potentially due to limited sample sizes. In the very late-stage subgroup (concurrent extrahepatic metastases and Vp4-type PVTT), the HAICAC regimen yielded a median OS of 27.4 months versus 19.4 months (HR:0.64, 95%CI:0.46-0.83, P<0.001) in patients with and without extrahepatic metastases, respectively. Corresponding median PFS were 11.2 months and 6.6 months (HR:0.59, 95%CI:0.39-0.89, P = 0.003). Kaplan-Meier curves of prognostic survival analysis for very late-stage subgroup is illustrated in **Figure 5**.

### 3.6 Safety

The adverse event profiles for both treatment groups are presented in **Table 4**. In the HAICAC group, the most frequently observed any grade adverse events were elevated AST (65.4%), hypoalbuminemia (64.2%), and elevated ALT (59.7%), with the most common grade 3–4 adverse events being leukopenia (17.6%), hand-foot syndrome (16.4%), and elevated AST (16.4%). The HAIC group showed a distinct pattern, with elevated AST (51.0%), nausea (46.2%) and hypoalbuminemia (42.0%) as the predominant any grade adverse events, while the most frequent grade 3–4 events were elevated AST (14.7%), elevated ALT (8.4%) and nausea (7.0%). In the HAICAC group, immune-related adverse events included hepatitis (20.8%; Grade 1–2: 19.5%, Grade 3–4: 1.3%), pneumonitis (3.1%), dermatitis (5.7%), and thyroiditis (1.3%). No cases of immune-related hypophysitis of any grade were observed. All immune-related adverse events were systematically monitored and either resolved with appropriate treatment or improved spontaneously. Although the incidence of immune-related adverse events (irAEs) was significantly higher in the combination therapy group compared to monotherapy, all events were manageable and no treatment-related mortality occurred during the study period.

**Table 4:** Treatment-related adverse events.

Adverse events	Grade 1/2			Grade 3/4		
	HAICAC (n=159)	HAIC (n=143)	P value	HAICAC (n=159)	HAIC (n=143)	P value
AEs-related treatment discontinuation, interruption or dose reduction						
HAIC interruption or dose reduction	15(9.4%)	17(11.9%)	0.489	32(20.1%)	37(25.9%)	0.235
Apatinib interruption or dose reduction	19(11.9%)	–	–	35(22.0%)	–	–
HAIC discontinuation	0(0%)	0(0%)	1.000	0(0%)	0(0%)	1.000
Apatinib discontinuation	5(3.1%)	–	–	12(7.5%)	–	–
Camrelizumab discontinuation	3(1.9%)	–	–	2(1.3%)	–	–
Apatinib-Camrelizumab discontinuation	2(1.3%)	–	–	2(1.3%)	–	–
Treatment-related AEs						
Hypertension	56(35.2%)	12(8.4%)	<0.001	19(11.9%)	2(1.4%)	<0.001
Diarrhea	44(27.7%)	21(14.7%)	0.009	8(5.0%)	5(3.5%)	0.512
Nausea	71(44.7%)	56(39.2%)	0.334	13(8.2%)	10(7.0%)	0.699
Vomiting	38(23.9%)	19(13.3%)	0.019	9(5.7%)	5(3.5%)	0.372
Weight loss	10(6.3%)	6(4.2%)	0.417	6(3.8%)	3(2.1%)	0.393
Fatigue	8(5.0%)	4(2.8%)	0.329	1(0.6%)	1(0.7%)	0.940
Inappetence	32(20.1%)	25(17.5%)	0.558	3(1.9%)	1(0.7%)	0.367
Fever	51(32.1%)	35(24.5%)	0.144	15(9.4%)	9(6.3%)	0.241
Abdominal pain	66(41.5%)	49(34.3%)	0.196	11(6.9%)	5(3.5%)	0.185
Neurologic toxicity	28(17.6%)	19(13.2%)	0.301	0(0%)	1(0.7%)	0.291
Rash	21(13.2%)	7(4.9%)	0.120	3(1.9%)	0(0%)	0.099
Hand-foot syndrome	38(23.9%)	11(7.7%)	<0.001	26(16.4%)	2(1.4%)	<0.001
Elevated ALT	74(46.5%)	45(31.5%)	0.007	21(13.2%)	12(8.4%)	0.180
Elevated AST	78(49.1%)	52(36.4%)	0.026	26(16.4%)	21(14.7%)	0.690
Anemia	62(39.0%)	34(23.8%)	0.005	17(10.7%)	6(4.2%)	0.034
Leukopenia	59(37.1%)	23(16.1%)	<0.001	28(17.6%)	7(4.4%)	<0.001
Neutropenia	47(29.6%)	16(11.2%)	<0.001	21(13.2%)	2(1.4%)	<0.001
Thrombocytopenia	55(34.6%)	35(24.5%)	0.055	20(12.6%)	8(5.6%)	0.037
Hypoalbuminemia	79(49.7%)	58(40.6%)	0.112	23(14.5%)	2(1.4%)	<0.001
Hyperbilirubinemia	45(28.3%)	31(21.7%)	0.185	21(13.2%)	3(2.1%)	0.001
Elevated creatinine	20(12.6%)	14(9.8%)	0.444	0(0%)	0(0%)	1.000
Proteinuria	11(6.9%)	8(5.6%)	0.636	2(1.3%)	1(0.7%)	0.625
Immune-related hepatitis	31(19.5%)	0(0%)	<0.001	2(1.3%)	0(0%)	0.178
Immune-related pneumonitis	5(3.1%)	0(0%)	0.032	2(1.3%)	0(0%)	0.178
Immune-related dermatitis	9(5.7%)	0(0%)	0.004	0(0%)	0(0%)	1.000
Immune-related thyroiditis	2(1.3%)	0(0%)	0.178	0(0%)	0(0%)	1.000
Immune-related hypophysitis	0(0%)	0(0%)	1.000	0(0%)	0(0%)	1.000

HAICAC, Hepatic arterial infusion chemotherapy combined with apatinib plus camrelizumab; HAIC, Hepatic arterial infusion chemotherapy; AEs: ALT, Alanine aminotransferase; AST, Aspartate aminotransferase.

The median treatment exposure duration was 8.2 months (range: 1.5–24.0) in the HAICAC group and 3.1 months (range: 1.0–18.5) in the HAIC group.In the HAICAC group, immune-related adverse events were managed according to institutional protocols based on CTCAE v5.0 grading. For immune-related hepatitis (Grade ≥2), camrelizumab was withheld, and oral prednisone was initiated at 1–2 mg/kg/day, with tapering upon resolution to Grade ≤1. Immune-related pneumonitis (Grade ≥2) mandated immediate interruption of camrelizumab and initiation of high-dose corticosteroids (e.g., methylprednisolone 1–2 mg/kg/day), with consideration of additional immunosuppressants in severe cases. Immune-related dermatitis and thyroiditis were managed supportively, using topical or systemic corticosteroids for severe dermatitis and hormone replacement for symptomatic hypothyroidism. For treatment-related liver function abnormalities, management followed CTCAE-based guidelines. For Grade ≥3 elevations in transaminases or bilirubin, HAIC was dose-reduced (5-FU dose adjusted to 300 mg/m² on day 1 plus 1800 mg/m² continuous infusion) and/or oral apatinib was modified (250 mg/day with 5-days-on/2-days-off or 7-days-on/7-days-off schedules), accompanied by active hepatoprotective therapy (e.g., magnesium isoglycyrrhizinate, glutathione, ursodeoxycholic acid). Treatment was resumed at the original or adjusted dose after liver function recovered to Grade ≤1. No irreversible liver failure events occurred. For myelosuppression, dose reduction of HAIC and/or apatinib was implemented, accompanied by supportive therapies such as granulocyte colony-stimulating factor (G-CSF), interleukin-11 (IL-11), or platelet transfusion as clinically indicated. For Grade 3–4 anemia, erythropoietin (EPO) or red blood cell transfusion was administered based on symptom severity. All hematologic toxicities resolved following treatment interruption, with no treatment-related mortality observed.

## 4 Discussion

HCC with Vp4 PVTT portends an exceedingly dismal prognosis, exhibiting a median OS of merely 6.5 months with standard sorafenib therapy. This multicenter retrospective study substantiates that the triple combination therapy comprising HAIC, apatinib, and camrelizumab exhibits remarkable therapeutic efficacy in Vp4 PVTT-associated HCC, with consistent outcomes both before and after matching. Following PSM adjustment, the HAIC combined with apatinib and camrelizumab regimen achieved an encouraging median OS of 24.1 months and median PFS of 7.0 months. Particularly noteworthy is the substantial enhancement in ORR demonstrated by the triplet therapy, showing 140.7% and 247.7% improvements over HAIC monotherapy for primary lesions (75.6% versus 31.4%) and PVTT (60.5% versus 17.4%), respectively. Furthermore, the triplet regimen attained an impressive DCR of 94.2%, representing a statistically significant advantage over the 57.3% DCR observed in the HAIC-alone cohort.

In the Asian region, the high tumor burden constitutes a defining characteristic of HCC, which inherently renders the purely systemic therapeutic approach of the BCLC staging system inapplicable, particularly for advanced stage patients with concurrent portal vein invasion (12). Conventional TACE is only feasible in a select minority of Vp4 patients with well-established portal venous collaterals, but it substantially elevates the risk of hepatic failure. Furthermore, two prior phase III trials demonstrated that transarterial radioembolization with yttrium-90 similarly failed to improve outcomes in PVTT patients, mirroring the limitations of sorafenib (13, 14). Additionally, surgical resection for HCC with Vp4 PVTT remains highly contentious and currently lacks robust high-level evidence to support its efficacy. As the sole remaining major locoregional therapeutic option, HAIC has demonstrated superiority in both therapeutic outcomes and adverse event profiles for Asian Vp4 populations. Owing to its proven efficacy in tumor control and downsizing across multiple clinical trials, HAIC has been endorsed by Chinese and Japanese guidelines as a first-line treatment for HCC with PVTT (15, 16). On the other hand, the epidemiology of HCC, particularly in East Asia, is predominantly driven by hepatitis B virus infection, while in Western regions, HCC is more commonly associated with hepatitis C virus infection, nonalcoholic steatohepatitis, or alcohol related liver disease. Consequently, the generalizability of the present findings to Western populations remains limited.

With the reports of numerous randomized trials on systemic treatment regimens in recent years, the comprehensive treatment based on HAIC has attracted attention and witnessed rapid progress. A previous randomized trial demonstrated that sorafenib combined with HAIC significantly improved survival outcomes compared to sorafenib monotherapy in patients with main trunk PVTT. For Vp4 PVTT, the addition of HAIC more than doubled both OS and PFS versus sorafenib alone, achieving a median OS of 13.6 months and a clinically meaningful median PFS of 6.8 months (17). An additional phase II trial evaluating the FOLFOX-HAIC and sorafenib combination regimen in major PVTT (Vp3/Vp4 type) similarly demonstrated positive PFS and ORR benefits (18). Following the subsequent report of IMbrave150 trial showing suboptimal efficacy (merely 7.6 months) of atezolizumab and bevacizumab in Vp4 PVTT. The CARES-310 trial which enrolled a high proportion of patients with portal vein involvement reported a groundbreaking median OS of 22.1 months, preliminarily demonstrating the potential efficacy of apatinib and camrelizumab combination in PVTT population (10). The recent TRIPLET clinical trial demonstrated that the triple-therapy regimen combining HAIC with apatinib and camrelizumab yielded favorable clinical outcomes in patients with BCLC stage C HCC. Although median OS was not reached, the combination achieved an encouraging median PFS of 10.4 months and a remarkable ORR of 77.1% as assessed by RECIST criteria. Notably, this trial enrolled 71.4% of patients with concurrent portal vein invasion. A multicenter retrospective study compared the efficacy of HAIC combined with lenvatinib and tislelizumab versus HAIC monotherapy for treating HCC with Vp4 PVTT (19). The HAIC monotherapy group achieved a median OS of 6.9 months, is comparable to our study findings. Notably, the combination therapy group demonstrated significantly improved outcomes, reaching a median OS of 23.2 months. However, this study’s limited sample size highlights the need for larger-scale clinical trials to validate these promising results.

The remarkable survival benefit of HAIC combined with apatinib and camrelizumab stems from their multifaceted synergistic mechanisms: (1) HAIC induces direct tumor cell DNA damage and apoptosis through localized delivery of high-concentration chemotherapeutic agents, while apatinib promotes tumor vascular normalization by inhibiting VEGFR-2-mediated angiogenic signaling, thereby enhancing drug delivery efficiency (20). (2) Immunogenic cell death triggered by HAIC releases abundant tumor-associated antigens and damage-associated molecular patterns, priming the tumor microenvironment for subsequent immunotherapy. Camrelizumab effectively activates antigen-presenting cells and cytotoxic T lymphocytes through PD-1/PD-L1 immune checkpoint blockade, establishing durable immune surveillance. Apatinib further modulates the tumor microenvironment to prolong the therapeutic window of immunotherapy (20). (3) Beyond its anti-angiogenic effects, apatinib significantly reduces tumor-associated macrophages, myeloid-derived suppressor cells and Tregs infiltration. This immunomodulation synergizes with camrelizumab checkpoint inhibition to promote effector T cell infiltration and functional activation (21). (4) VEGF pathway inhibition downregulates PD-L1 expression, enhancing PD-1 inhibitor sensitivity. Concurrently, chemotherapy-activated innate immune pathways potentiate interferon signaling, creating signal transduction-level synergy with checkpoint inhibitors (22).

The clinical treatment of Vp4 PVTT is governed by a complex interplay of tumor biological behavior and underlying host factors. Our analysis identified ECOG performance status, ALBI grade and treatment modality as independent determinants of overall survival, while PFS was significantly influenced by ECOG status, serum AFP levels, presence of extrahepatic metastases, and therapeutic regimen. Of particular clinical relevance, AFP elevation serves as a quantitative biomarker of tumor aggressiveness, with markedly elevated titers demonstrating strong correlations with increased tumor burden, extensive vascular invasion, and metastatic dissemination which collectively contributing to an accelerated disease trajectory (23). Extrahepatic metastasis also constitutes one of the most aggressive manifestations of advanced HCC progression, accelerating disease trajectory while precluding eligibility for curative-intent interventions, ultimately culminating in dismal prognosis. Additionally, the ALBI scoring system offers superior objectivity compared to conventional Child-Pugh classification by eliminating subjective parameters such as ascites and encephalopathy, thereby providing a more reliable assessment of hepatic function (24). Importantly, while liver function per se does not directly modulate neoplastic progression, preserved hepatic reserve critically determines treatment tolerability and maintenance of stable liver function enables uninterrupted therapeutic delivery, which in turn translates to meaningful survival advantages for this challenging patient population (25).

Two previous phase III trials have conclusively demonstrated that HAIC yields superior tumor regression and subsequent conversion resection rates compared to standard sorafenib therapy or TACE (5, 26), ultimately translating into significant survival benefits. This therapeutic advantage is particularly pronounced in patients with high tumor burden and more advanced disease stages. In this study, the HAICAC regimen demonstrated significantly higher ORR and DCR in both primary tumors and PVTT, underscoring its efficacy in controlling disease progression and inducing tumor and PVTT regression, even in aggressive Vp4 PVTT subtypes. Subgroup analyses demonstrated consistent OS benefits across all Vp4 PVTT subgroup treated with the triplet regimen, regardless of tumor size, multifocality, or extrahepatic metastasis status, underscoring the broad clinical applicability of this therapeutic approach. However, PFS improvements were not observed in non-cirrhotic patients, those with Child-Pugh B liver function, or tumors<7cm, likely due to limited subgroup sample sizes introducing statistical bias. Strikingly, the regimen conferred robust survival advantages in extremely advanced HCC cases featuring concurrent Vp4 PVTT and extrahepatic metastases, with median OS and PFS reaching 19.4 months and 6.6 months, respectively.

In terms of safety profile, transient hepatic function impairment was observed with HAIC either alone or in combination with apatinib and camrelizumab, with all cases of liver function decline demonstrating rapid recovery following appropriate interventions. The augmented hepatotoxicity associated with combination therapy was unavoidable, underscoring the critical importance of long-term hepatic monitoring and timely clinical intervention. Notably, the HAICAC regimen exhibited a 40.3% incidence of hand-foot syndrome, which was significantly higher than that observed in other targeted-immunotherapy combinations; this difference was primarily attributable to apatinib administration. Furthermore, camrelizumab incorporation inevitably induced myelosuppression and immune-related hepatitis, necessitating regular hematological surveillance. Although the combination of HAIC with apatinib and camrelizumab demonstrated significantly increased incidence rates of both hepatotoxicity and immune-related adverse events compared to HAIC monotherapy, all grade 3–4 adverse reactions were effectively managed without any grade 5 treatment-emergent adverse events, indicating that the incremental toxicities associated with apatinib and camrelizumab are clinically manageable.

This study has several limitations that warrant acknowledgment. Firstly, as a retrospective analysis, although PSM was employed to mitigate intergroup disparities, inherent heterogeneity in clinical data remains unavoidable. Secondly, the enrolled HCC cases were exclusively hepatitis B virus related. thus, the generalizability of findings to other etiologies, such as hepatitis C virus infection and alcohol-related liver disease prevalent in Western regions, requires further investigation. Thirdly, our safety analysis reports crude incidence rates of adverse events. Given the significantly longer treatment exposure duration in the HAICAC group due to superior survival, direct comparison of toxicity incidence may be subject to detection bias, and future studies incorporating exposure-adjusted analyses would be valuable. Furthermore, while using the time of diagnosis as the starting point for survival analysis reflects the complete clinical pathway from diagnosis to treatment in real-world practice, it also includes intervals attributable to non-treatment factors such as treatment delays and multidisciplinary decision-making. This may introduce certain limitations to the precise assessment of survival time. Additionally, imbalanced sample sizes across certain subgroups may introduce potential bias into the subgroup analyses. Consequently, large-scale randomized trials are warranted to validate these findings.

## 5 Conclusion

The combined regimen of HAIC and apatinib-camrelizumab demonstrates robust efficacy in controlling disease progression and reducing intrahepatic and portal venous tumor burden in HCC patients with Vp4 PVTT. This therapeutic approach significantly improves survival outcomes, enables long-term survival, and maintains a tolerable safety profile, establishing it as a promising and viable treatment option for Vp4 PVTT-associated HCC.

## Data Availability

The raw data supporting the conclusions of this article will be made available by the authors, without undue reservation.
