# Supplementary material for: Hepatic arterial infusion chemotherapy combined with apatinib plus camrelizumab for advanced hepatocellular carcinoma with type Vp4 portal vein tumor thrombosis: a multicenter propensity score-matching analysis
Source: Front Immunol. 2026 Feb 17;17:1742116. doi: 10.3389/fimmu.2026.1742116 (PMC12953477; doi:10.3389/fimmu.2026.1742116)
Supplement: Supplementary file 1 [file Supplementaryfile1.docx]

**Hepatic Arterial Infusion Chemotherapy combined with Apatinib plus Camrelizumab for Advanced Hepatocellular Carcinoma with Type Vp4 Protal Vein Tumor Thrombosis: A Multicenter Propensity Score-Matching Analysis**

**This supplementary material includes:**

**HAIC procedure**

All vascular interventional treatments were performed by two or more experienced interventional imaging physicians under digital subtraction angiography (DSA) guidance achieving technical success.

**HAIC** procedure**:** The procedure for **Hepatic artery catheterization** are as follows: Following femoral artery puncture via the modified Seldinger technique, a 5F vascular sheath was inserted. Through this sheath, a 5F Yashiro catheter (Terumo, Tokyo, Japan) was introduced for angiography of the superior mesenteric artery and celiac trunk in sequence. This was aimed at precisely discerning the origins of the intra- and extra-hepatic arteries that supply the tumor. Subsequently, a 2.7Fr microcatheter system (Terumo Corporation, Tokyo, Japan) was placed.

mFOLFOX6 - HAIC protocol: Oxaliplatin (85 mg/m^2^) was given via 2-hour infusion; leucovorin (400 mg/m^2^) was administered via 2-hour infusion; 5-FU (400 mg/m^2^) was given as a bolus, followed by continuous infusion of either 2400 mg/m^2^ over 46 hours or 1200 mg/m^2^ over 23 hours. HAIC was repeated every 3-4 weeks. Dose adjustments were implemented when persistent or severe treatment-related adverse reactions occurred, with therapy resumed once the patient condition stabilized. The HAIC regimen was repeated every four weeks. Post-treatment, a full abdominal enhanced CT scan was conducted every eight weeks to assess treatment efficacy.
